# Development and *In Vivo* Evaluation of Ziyuglycoside I–Loaded Self-Microemulsifying Formulation for Activity of Increasing Leukocyte

**DOI:** 10.1208/s12249-019-1313-3

**Published:** 2019-02-05

**Authors:** Yongai Xiong, Ya Zou, Li Chen, Yingshu Xu, Sen Wang

**Affiliations:** School of Pharmacy, Zunyi Medical University, West No. 6 Xuefu Road, Xinpu District, Zunyi, 563000 Guizhou People’s Republic of China

**Keywords:** ziyuglycoside I, SMEDDS, bioavailability, solubility

## Abstract

Ziyuglycoside I (ZgI), a major effective ingredient of *Sanguisorba officinalis* L, has shown good activity in increasing leukocyte of myelosuppression mice. However, oral ZgI therapy has been deterred by poor bioavailability because of its low aqueous solubility and permeability. Our study was to develop ZgI-loaded self-microemulsifying drug delivery system (SMEDDS) and evaluate its intestinal absorption, and pharmacokinetic and pharmacodynamic activity for increasing leukocyte. The formulation was designed and optimized by measuring the equilibrium solubility of ZgI in different vehicles and the pseudoternary phase diagram. Further, morphology, particle size, stability, *in vitro* release, *in situ* single-pass intestinal perfusion (SPIP), *in vivo* activity, and *in vivo* pharmacokinetic (PK) of ZgI-SMEDDS were charactered or studied. Optimized formulations for *in vitro* dissolution were Obleique CC497, Tween-20, and Transcutol HP with a proportion of 0.25/0.45/0.30 *via* D-optimal mixture design. Results showed that the solubility of ZgI was enhanced up to 23.93 mg/g and its average particle size was 207.92 ± 2.13 nm. The release of ZgI had been greatly improved by the SMEDDS. In SPIP, the intestinal absorption of SMEDDS was much better than plain ZgI. In PK, we found the oral bioavailability of ZgI-SMEDDS was 6.94-fold higher absolute bioavailability (21.94 ± 4.67) % than ZgI (3.16 ± 0.89) %. The most important was that the mice WBC of ZgI-SMEDDS group was significantly higher than that of the ZgI group. Our study suggested that SMEDDS could increase the solubility of ZgI, which was beneficial to improve oral bioavailability and enhance biological activity.

## INTRODUCTION

Clinically, the bone marrow of cancer patients are severely damaged by multiple chemoradiotherapy or large doses of chemotherapy drugs, which usually lead to toxic effects(1,2), such as the myelosuppression which is the most frequent and serious one. Once patients suffer from myelosuppression, they have to bear great pain and heavy economic burdens which are caused by leukopenia, neutropenia, anemia, and so on (3). What’s more, doctors have to change the CRT schedule or reduce the dose of chemotherapeutic agents. So, protecting the bone marrow and increasing hemocyte (especially the WBC) counts of peripheral blood are very important for tumor patients to undergo chemoradiotherapy and improve their life quality.

**Fig. 1:**
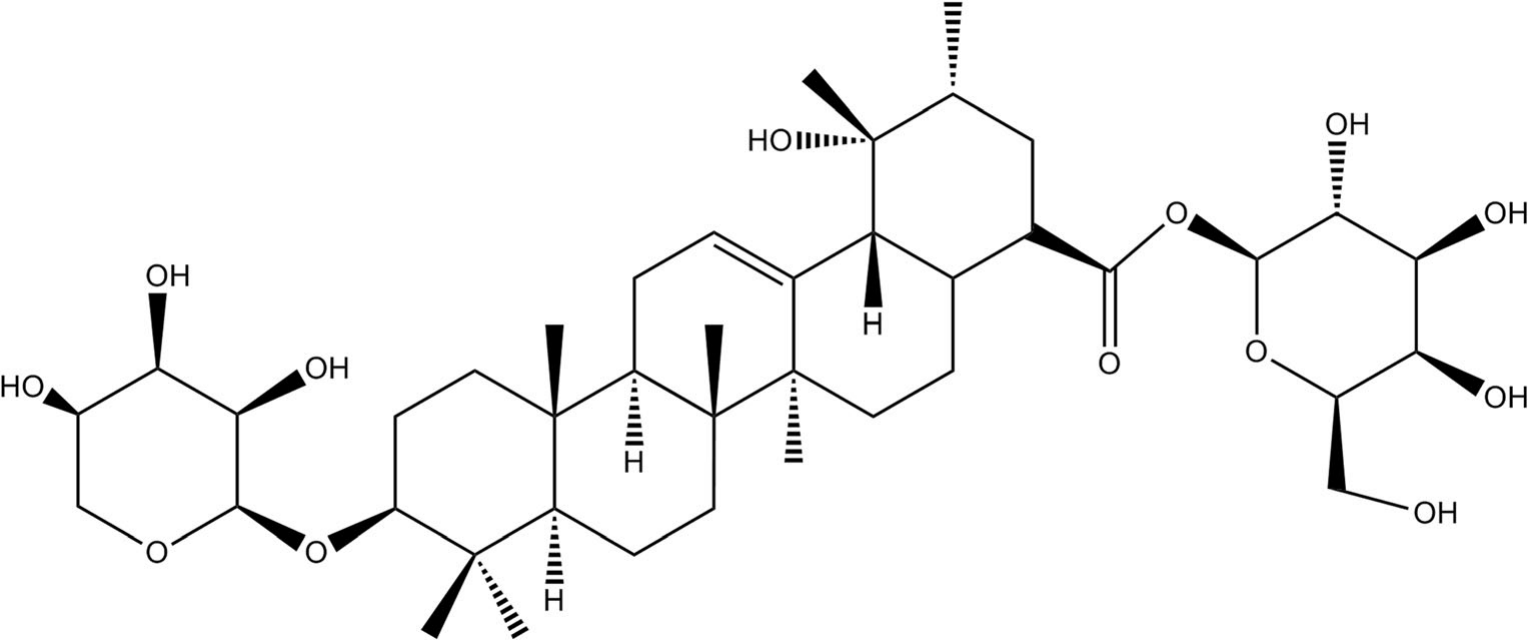
The chemical structure of ZgI

Our previous research showed that ZgI was the main active ingredient of *Sanguisorba officinalis* (4–6). It is a kind of pentacyclic triterpenoid saponin, as its chemical structure is shown in Fig. 1. It can promote the proliferation of hematopoietic stem cells (HSCs) in mouse bone marrow and increase the number of hemocytes in mouse peripheral blood, which is important to alleviate the side effects of myelosuppression induced by anticancer drug or radiation (7–9). ZgI is type 4 drug in BCS; it is hardly soluble in water, which has become a major factor limiting its oral bioavailability.

The self-microemulsifying drug delivery system (SMEDDS) is a transparent, homogeneous solution composed of an oil phase, a surfactant, a cosurfactant, and a drug. It can spontaneously emulsify to form microemulsion with particle size 10–100 nm in the aqueous phase at ambient temperature and mild agitation (10), which is important to enhance the solubility and oral bioavailability of poorly water-soluble or fat-soluble drugs (11,12). SMEDDS can be absorbed by the lymphatic pathway, which can reduce the first-pass metabolism of drugs in the liver and the decomposition of drugs by gastrointestinal enzymes (13,14). Part of the components of SMEDDS can inhibit the catalysis of intestinal cytochrome P450 and the efflux of P-gp to drugs, such as Cremophor EL, Tween-80, caprylic acid and citric acid, Labrasol, Myrj 52, Brij 30, polyethylene glycol (PEG) 400, and PEG 20000 (15–17).

In this paper, we tried to develop an oral self-microemulsion of ZgI, which may be effective to improve the solubility, *in vivo* release, and gastrointestinal absorption of ZgI. In short, we intended to enhance the bioavailability and activity of ZgI for increasing WBC.

## MATERIALS AND METHODS

### Chemicals and Reagents

ZgI was extracted and purified in the lab. ZgI Reference (lot number MUST-17022502, mass fraction 99.47%) was purchased from Chengdu Pfizer Biotech Co., Ltd., China. Labrafil M 1944CS, Obleique CC497, Labrasol, and Transcutol P were purchased from France Jiafa Lions; medium-chain triglycerides (MCT) Mic acid ester, isopropyl myristate (IPM), oleic acid, ethyl oleate, and isopropyl palmitate (IPP) were purchased from Shanghai Chuxing Chemical Co., Ltd.; castor oil, Tween-20, Tween-80, Tween-85, PEG-600,PEG-200, and PEG-400 were purchased from Chengdu Kelon Chemical Reagent Factory; and all other chemicals and reagents used were of HPLC grade.

### Animals

SPF Kunming (KM), ♀/♂, weighing 20–25 g, were purchased from Laboratory Animal Center of Zunyi Medical University (certificate No. SCXK(Qian) 2013–0006). SPF rats, ♀/♂, weighing 220 ± 20 g were purchased from the Third Military Medical University of the People’s Liberation Army (Grant No. scxk(army)2012-0011). Mice or rats were housed in normal cages and 12-h light/dark cycle (6.30 am to 6.30 pm).

### Preparation of SMEDDS

#### Determination of Equilibrium Solubility

The equilibrium solubility of ZgI was measured in various oils, surfactants, and cosurfactants. ZgI was excessively added into gcentrifuge tubes containing 2 g of a vehicle (oil, surfactant, or cosurfactants), and the mixtures were stirred using thermostatic oscillator for 72 h at 37°C and 150 r/min. After shaking, the mixtures were centrifuged at 8000 rpm for 8 min and supernatants were filtrated using Millipore filter (0.22 μm). The ZgI concentration was determined using a HPLC.

#### Preliminary SMEDDS Preparation

Solubility and pseudoternary phase diagram are usually used to select optimal formulations. According to the equilibrium solubility results of ZgI in different vehicles, the desired oil, surfactant, and cosurfactant which had the highest solubility to ZgI would be selected. Then the proportion range of oil, surfactant, and cosurfactant were set according to lipidic formulation classification system (oil, 10–50%; surfactant and cosurfactant, 0–80%) and the solubility test in SMEDDS and superimposed ternary phase diagrams. So the contents of the surfactant, cosurfactant, and oil were in the ranges of 10–50% *w*/*w* of each excipient (18). The solubility of ZgI in various mixtures was investigated. The resulting mixtures were subsequently titrated with water under moderate agitation at ambient temperature. SMEDDS were characterized on the basis of appearance, emulsification time, droplet size, and solubility of ZgI. All formulations were prepared in triplicate. The pseudoternary phase diagrams were designed using OriginPro10 for Windows (OriginLab Corporation, USA).

#### Design of Experiments

The D-optimal mixture design of a three-factor, two-level SMEDDS formulation was performed using design of experiments (DoE) to determine the individual effects and interaction effects of excipient concentration on the solubility of ZgI in SMEDDS and the particle size of dilute SMEDDS. Obleique CC497 (factor A) concentrations ranged from 5 to 25%, Tween-20 (factor B) concentrations ranged from 45 to 65%, and Transcutol HP (factor C) concentrations ranged from 10 to 30%. The Design Expert 8.0.4.1 software was used to study the effect of these formula variables (factors A–C) on the dependent variable (*Y*_1_: solubility of ZgI in SMEDDS and *Y*_2_: particle size of diluted SMEDDS). Twenty experiments were randomly designed by software and repeated two times to improve predictability. All experiments were kept at 100% batch size.

#### Characterization of SMEDDS

The particle size, polydispersity index (PDI), and zeta potential of optimized SMEDDS were measured by dynamic light scattering using a 90Plus PALS laser particle size analyzer (Brughaven, USA). SMEDDS diluted 200-fold was transferred to a standard cuvette at 25°C, and measured with a fixed angle of 90°. The software was used to analyze the average particle size, PDI, and zeta potential. Each sample was analyzed in triplicate.

#### Stability Test

The ZgI-loaded SMEDDS were put in small plastic bottles and stored in a stability chamber (SHH-500SD, ShanghaiZuocheng Co., Ltd., Shanghai, China). 25°C/60% RH and 40°C/75% RH for 3 months were set to study the stability. Samples were taken out at 0, 1, 2, and 3 months for analysis, respectively (19).

#### Drug-Excipients Compatibility Studies

Optimized liquid SMEDDS were converted in solid by using the lyophilizer (Labconco, FreeZone5L, USA) and further characterized by Fourier transform infrared (FTIR) spectroscopy, differential scanning calorimetry (DSC), transmission electron microscopy (TEM), and particle size analysis.

##### FTIR Spectroscopy

The IR spectra were recorded on the FTIR (Thermo Scientific, FTIR model IS5, USA) by using a potassium bromide pressed-disk method. The scanning range was 450–4000 cm^−1^.

##### DSC Analysis

The physical state of ZgI in SMEDDS was characterized by the DSC (Seiko, EXSTAR 6000, Japan). The samples (about 5.00 mg) were placed in standard aluminum pans and the air was used as N_2_. All samples were scanned at a temperature ramp speed of 20°C/min and the heat flow was set from 25 to 250°C.

##### TEM

ZgI-SMEDDS were diluted 200 times with triple distilled water and 2% phosphotungstic acid solution was added and mixed by slightly shaking for 1 min. One drop of diluted sample was deposited on a carbon-coated copper grid and observed under the TEM.

#### *In Vitro* Drug Release

ZgI SMEDDS and ZgI were poured into No.0 capsule, respectively. The drug release was performed using shaking water bath operated at (37.0 ± 0.5) °C at 50 ± 1 strokes/min in 500 mL of dissolution medium containing 0.1% *w*/*v* hydrochloric acid. Samples were taken at 10, 20, 30, 45, 60, 90, and 120 min at different time intervals and maintained sink condition with 1 mL of fresh medium, then filtered by 0.22 μm microporous membrane. The cumulative percentage drug release was analyzed by the validated HPLC method.

#### In situ Single Pass Intestinal Perfusion

The single-pass intestinal perfusion (SPIP) studies of ZgI-SMEDDS were carried out in Sprague Dawley (SD) rats. Twelve rats were randomly divided into two groups (ZgI and ZgI-SMEDDS) of six rats each and were housed in IVC animal house and lighted on a regular 12-h light-dark cycle. The methods for the SPIP studies were carried out according to references (20,21). Briefly, 10% chloral hydrate (4.0 mL/kg) was i.p-injected to anesthetize rats; then, rats were placed on a heating operating table to maintain a body temperature of 37°C. After the loss of pain reflex, rats’ abdomen were opened and a midline longitudinal incision (3–4 cm) was carefully made; then, a 10–15 cm of the jejunum was isolated and catheterized at both ends with plastic tubing. Thirty-seven degree Celsius saline solution was firstly used to clear the segment for 15 min, then drug perfusion solution which contained100 mg/mL ZgI (in ZgI Krebs-Rings solution and ZgI-SMEDDS) and 100 μg/mL phenol red (marker) was pumped into jejunum *via* peristaltic pump (Shanghai Huxi Analytical Instrument Factory Co., Ltd.) at a rate of 0.2 mL/min. When the steady state reached after about 30 min, perfused samples from the outlet should be collected on ice every 15 min for 150 min. Samples were immediately stored at − 20°C until analysis. At the end of the experiment, the perfused intestinal segment was measured without stretching and finally the animal was euthanized. The melted samples were centrifuged at 10,000 rpm for 10 min. A 10 μL aliquot of the supernatant was injected into the HPLC system. The absorption rate constant (*K*_a_), apparent permeability coefficients (*P*_app_) and drug absorption percentage (*A*%)were calculated as the following formulas:

$$ {V}_{\mathrm{in}}={m}_{\mathrm{in}}/{\rho}_{\mathrm{in}}\ {V}_{\mathrm{out}}={m}_{\mathrm{out}}/{\rho}_{\mathrm{out}} $$

$$ A\%=\left(1-\frac{C_{\mathrm{out}}}{C_{\mathrm{in}}}\times \frac{Q_{\mathrm{out}}}{Q_{\mathrm{in}}}\right)\times 100\% $$

$$ {K}_a=\left(1-\frac{C_{\mathrm{out}}}{C_{\mathrm{in}}}\times \frac{Q_{\mathrm{out}}}{Q_{\mathrm{in}}}\right)\times \frac{Q}{V} $$

$$ {\mathrm{P}}_{app}=\frac{-Q\times \ln \left[\frac{C_{out}\times {Q}_{out}}{C_{in}\times {Q}_{in}}\right]}{2\pi \mathrm{rl}} $$

where *m*_in_ is the weight of the inlet solution, *m*_out_ is the weight of the exiting solution, *ρ*_in_ is the density of the inlet solution, *ρ*_out_ is the density of the exiting solution, *V*_in_ is the volume of the inlet solution, *V*_out_ is the volume of the exiting solution, *Q* is the flow rate (mL/min) of the inlet solution, *C*_in_ is the concentration (μg/mL) of the drug in the inlet solution, *C*_out_ is the concentration (μg/mL) of the drug in the exiting solution, *V* is the volume of the perfused segment (mL), *l* is the length of the intestinal segment (cm), and *r* is the radius of the intestinal segment (cm).

The *A*%, *K*_a_, and *P*_app_ values ​of ZgI were calculated from 45 to 75 min to 165 to 195 min in each intestine segment, and the mean value of *A*%, *K*_a_, and *P*_app_ of ZgI at five time periods were calculated. The average value was used as the actual *A*%, *K*_a_, and *P*_app_ value of the intestine segment. SPSS22.0 statistical software was used to analyze the results by one-way ANOVA.

#### *In Vivo* Activity Study

Mice were randomized according to their body weight into four experimental groups after 3 days of acclimatization: control group, cyclophosphamide (CP) group, ZgI-SMEDDS group (10 mg/kg, respectively), and ZgI group (10 mg/kg, respectively). Except the control group, mice in other groups were injected intraperitoneally with a single dose of CP (150 mg/kg, 0.2 mL/10 g) on day 0 and the control group received an equal volume of saline correspondingly. Immediately after CP treatment, mice were orally administrated ZgI-SMEDDS and ZgI. The normal and CP groups received an equivalent volume of distilled water, once a day for six consecutive days. On day 6, the blood from eye vein was collected and placed into a lavender top collection tube containing EDTA and kept at ambient temperature. WBC was measured by a cell counter (Bayer Advia 120, Sysmex, Kobe, Japan) at times up to 12 h after blood collection. The experimental protocol was approved by the Animal Ethics Committee for animal experimentation of Zunyi Medical University (Ethic numbers (2018)2-030).

#### Pharmacokinetic Studies

The PK studies of ZgI were carried out in SD rats (male and female). One week after the SD rats were fed adaptively, they were randomly divided into three groups according to their body weight, *i*.*e*., the Zg I group, the ZgI-SMEDDS group, and the ZgI-intravenous (i.v) group each containing six animals. After being fasted for 12 h, the ZgI group of animals received oral Tween-20 solution of ZgI (20 mg/kg) and ZgI-SMEDDS (25 mg/kg, equivalent to free ZgI). Animals in the ZgI-i.v group were administered with i.v Tween-20 solution of ZgI (2 mg/kg, equivalent to free ZgI). Blood specimens were collected at the time of 0.1 h, 0.25 h, 0.5 h, 1.0 h, 2.0 h, 4.0 h, 6.0 h, 8.0 h, 10.0 h, 12.0 h, and 24.0 h after administration (22); 0.5 mL of blood sample of each animal was collected from the fundus veins into heparinized centrifuge tubes and was centrifuged at 5000 rpm for 10 min. The plasma kept at −80°C until assay.

#### Analytical Method for ZgI Quantification

Agilent 1260 HPLC. The column was a Unitary C_18_ column (250 mm × 4.6 mm, 5 μm), the mobile phase was acetonitrile-water (32:68), the volume flow rate was 1 mL/min, the column temperature was 30°C, the detection wavelength was 203 nm, and the injection volume was 10 μL (23).

#### Statistical Analysis

The data are presented as means ± S.E.M. Kolmogorov-Smirnov’s test was used to evaluate normality of the data distribution. Data was then analyzed by an independent *t* test or a one-way ANOVA followed by Tukey’s post hoc test. Statistical significance was considered at *p* values < 0.05.

## RESULTS

### Solubility of ZgI in Different Excipients

In order to increase the solubility of ZgI in SMEDDS, the solubility of ZgI in various excipients was investigated to select excipients with greater solubilization potential. The solubility of ZgI in various excipients is shown in Table I. The maximum solubility of ZgI in mg/g was observed in Transcutol HP (9.75 mg/g), followed by Tween-20 (4.23 mg/g), Obleique CC497 (2.87 mg/g), and Oleic acid (2.63 mg/g). According to the results of saturated solubility, further research will be conducted on the excipients with greater solubilization potential.

**Table I:** Results of the Solubility of ZgI in Various Excipients ($\overline{x}$ ± *S*, *n* = 3)

Excipients		Solubility (mg/g)
Oil	Labrafil M 1944CS	1.79 ± 0.18
	Obleique CC497	2.87 ± 0.57
	Castor Oil	0.88 ± 0.09
	IPP	0.56 ± 0.03
	Oleic Acid	2.63 ± 0.15
	MCT	0.45 ± 0.01
	IPM	0.52 ± 0.01
	Ethyl Oleate	0.44 ± 0.01
Surfactant	Labrasol	1.40 ± 0.14
	Tween-20	4.23 ± 0.33
	Tween-85	1.43 ± 0.13
	Tween-80	1.55 ± 0.19
Cosurfactant	PEG-400	8.58 ± 0.59
	PEG-600	7.91 ± 0.72
	PEG-200	9.13 ± 0.61
	Transcutol P	9.75 ± 0.53

### Optimization of Excipients for Preparation of SMEDDS

**Fig. 2:**
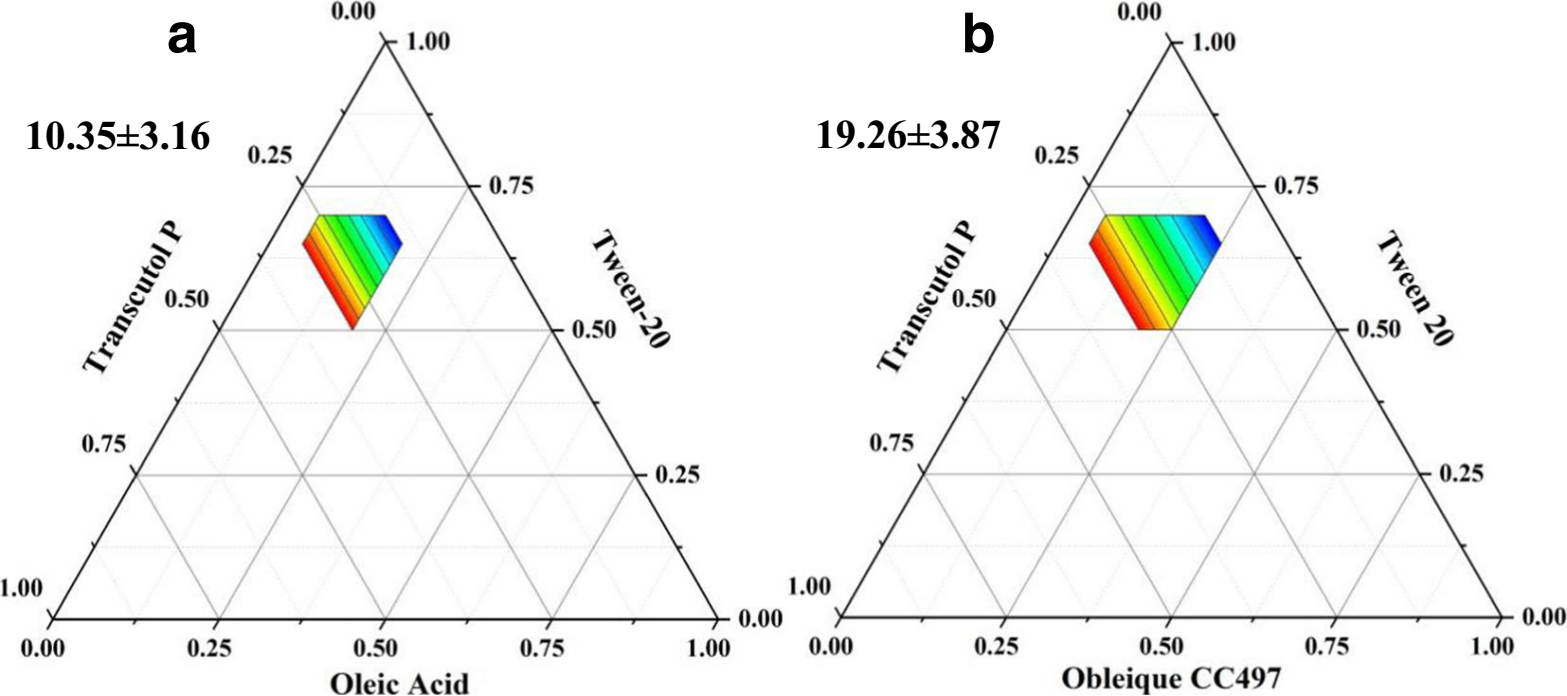
Ternary phase diagram. **a** Oleic acid-Tween-20-Transcutol P. **b** Obleique CC497-Tween-20-Transcutol P. The colorized regions represent the microemulsion phase

Before using DoE, we should select the most appropriate oil phase by drawing a ternary phase diagram and further confirm the ratio of the prescription. The ternary phase diagram was constructed to identify the desired SMEDDS regions. Finally, the size of microemulsion area was calculated to determine which the best oil phase is. As shown in Fig. 2. It was found that the areas of monophasic transparent formulations using Oleic acid and Obleique CC497 were10.35 ± 3.16 and19.26 ± 3.87% of the total phase diagram, respectively. The results suggested that Obleique CC497 created larger monophasic area in the phase diagrams. So Obleique CC497, Tween-20, and Transcutol P were used to make ZgI-SMEDDS.

### D-Optimal Mixture Design

The weight of Obleique CC497(*X*_1_/g), Tween-20(*X*_2_/g), and Transcutol P(*X*_3_*/g*) was chosen as the factors; the drug loading rate (*Y*_1_*/*mg g^−1^) and particle size(*Y*_2_/nm) were chosen as the evaluation index. The response data for all experimental runs of the D-optimal mixture design are shown in Table II. An analysis of variance (ANOVA) was used to estimate the significance of the effect of each variable and its interaction. Four kinds of mathematical models were used to perform regression fitting and analysis. The standard variance, complex correlation coefficient, predictive compound correlation coefficient, and adjusted correlation coefficient of the regression model were used as comprehensive indicators to judge and select the best regression model among the four mathematical models. The results are shown in Table III.

**Table II:** D-Optimal Mixture Design and Results

No.	*X*_1_/g	*X*_2_/g	*X*_3_/g	*Y*_1_/mg g^−1^	*Y*_*2*_/nm
1	0.25	0.57	0.22	24.09	208.80
2	0.05	0.53	0.18	23.21	105.38
3	0.05	0.65	0.30	24.54	122.12
4	0.13	0.45	0.22	22.10	164.08
5	0.25	0.57	0.22	25.78	211.02
6	0.25	0.57	0.22	26.17	251.97
7	0.05	0.52	0.30	22.88	155.35
8	0.25	0.45	0.30	23.93	207.92
9	0.13	0.45	0.22	22.98	142.73
10	0.23	0.55	0.12	20.78	255.37
11	0.17	0.53	0.30	23.34	197.59
12	0.17	0.64	0.18	23.26	147.95
13	0.13	0.45	0.22	22.15	174.81
14	0.05	0.45	0.10	14.68	281.48
15	0.05	0.65	0.17	23.46	102.58
16	0.13	0.57	0.10	22.96	508.41
17	0.25	0.65	0.10	18.21	268.24
18	0.18	0.65	0.30	22.79	110.79
19	0.25	0.45	0.10	23.38	250.64
20	0.13	0.57	0.10	21.66	469.49

**Table III:** The Regression Analysis Results of the Experiment

Response	Model	*S*	*r*^2^	Adjusted *r*^2^	Predicted *r*^2^	Remark
*Y*_1_	Linear	1.67	0.5741	0.4943	0.2519	suggest
	Quadratic	1.56	0.6953	0.5547	− 0.1852	–
	Special	1.24	0.8526	0.7200	− 0.1751	suggest
	Cubic	0.58	0.9870	0.9381	–	–
*Y*_2_	Linear	91.28	0.4058	0.2944	0.1175	–
	Quadratic	98.89	0.4333	0.1717	− 0.4305	–
	Special	65.86	0.8066	0.6326	0.2692	Suggest
	Cubic	23.07	0.9905	0.9549	–	–

Fitting model regression

Equation: *Y*_1_ = 24.40 + 0.75*A* + 1.18*B* + 1.45*C* − 1.14AB − 0.52 AC − 0.89 BC − 0.19*A*^2^ − 1.09*B*^2^ − 1.68*C*^2^

*Y*_*2*_ = 238.38 + 17.41*A* − 8.63*B* − 85.22*C* − 16.65AB + 31.95 AC − 18.51 BC − 58.42*A*^2^ − 85.79*B*^2^ + 107.10*C*^2^

#### Influence of Formulation Variables on Response Y_1_ (Solubility of ZgI)

**Fig. 3:**
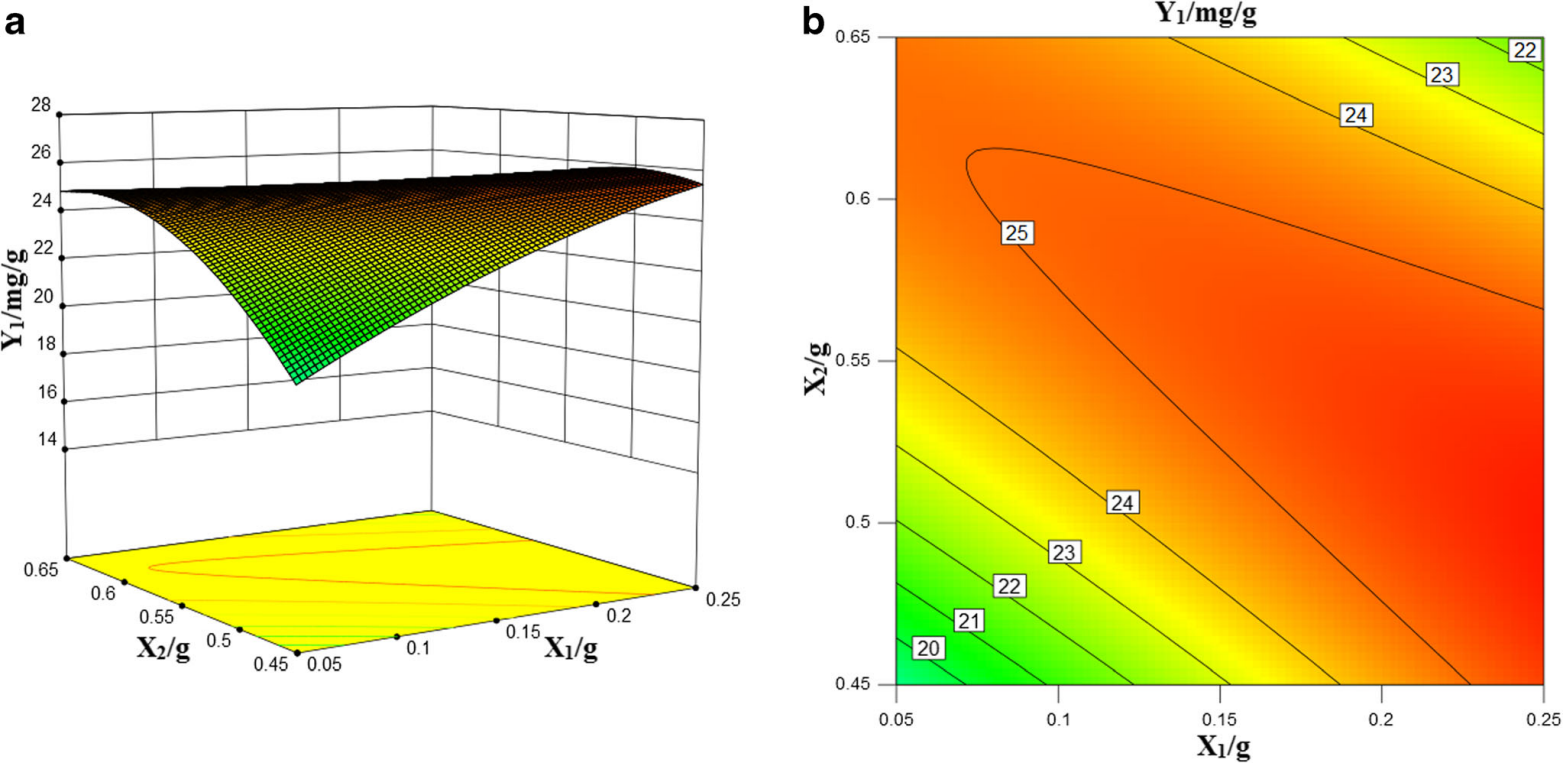
Drug loading 3D stereogram (**a**) and 2D contour map (**b**)

The positive sign in the polynomial regression equation indicates the synergy effect, and the negative sign indicates the antagonistic effect. In the special cube model that responds to *Y*_1_, three factors alone (Transcutol HP, Obleique, Tween-20) have positive effects, of which Obleique has a significantly lower effect. The complex correlation coefficient of the *Y*_1_ response value is higher, which indicated that the regression model was fitted well and the regression equation had a good representation and could accurately predict the actual situation. The 3D stereogram and 2D contour of *Y*_1_ response values are shown in Fig. 3.

#### Influence of Formulation Variables on Response Y_2_ (Droplet Size upon Dilution)

**Fig. 4:**
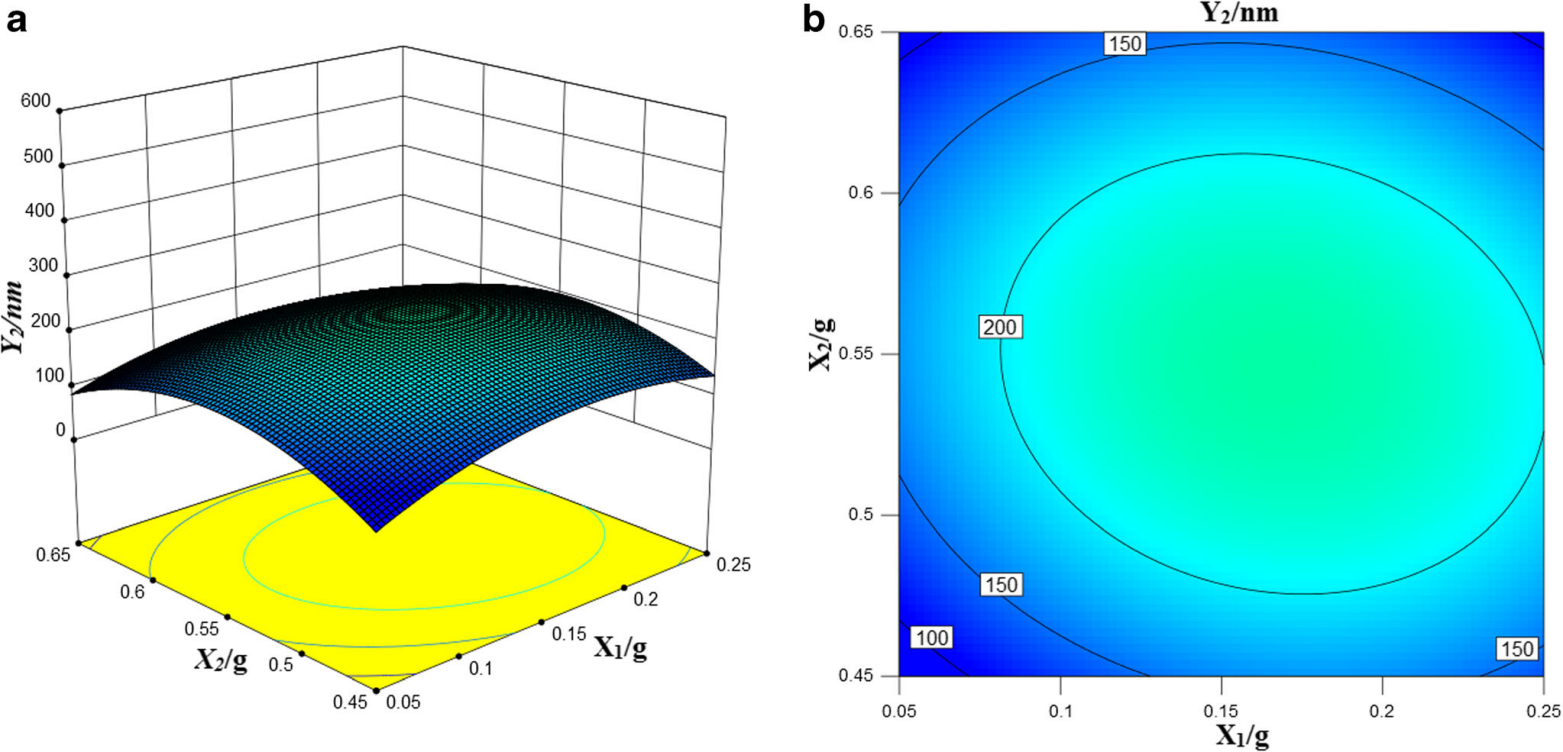
Particle size 3D stereogram (**a**) and 2D contour map (**b**)

In the special cubic model response to *Y*_2_ other than Obleique CC497, the remaining two factors alone have a negative effect on droplet size after dilution. That is, as the concentration increases, the droplet size decreases. The complex correlation coefficient of the *Y*_2_ response value is higher, which indicated that the regression model was fitted well and the regression equation has a good representation and can accurately predict the actual situation. The 3D stereogram and 2D contour of *Y*_2_ response values are shown in Fig. 4.

#### Optimized Formulation Verification

From the D-optimal mixture design result, we got the optimal formulation of SMEDDS, which was composed of CC497-Tween-20-Transcutol P (0.25:0.45:0.30). Then the optimized formulation was repeated three times. The polydispersity index of the prescription was less than 0.3, so it could meet the stability requirements of the preparation.

### Characterization of SMEDDS

The optimal formulation was diluted 200 times with water in a volumetric flask under stirring conditions. The zeta potential, droplet size, and polydispersity index were measured with particle size analyzer (Mastersizer 3000, Malvern Panalytical Co, Ltd., UK). The average droplet size of SMEDDS was 207.92 ± 2.13 nm, the polydispersity index was 0.264 ± 0.015, and the zeta potential was − 38.84 ± 0.18 mV.

### Result of ZgI-Excipients Compatibility Studies

#### FTIR

**Fig. 5:**
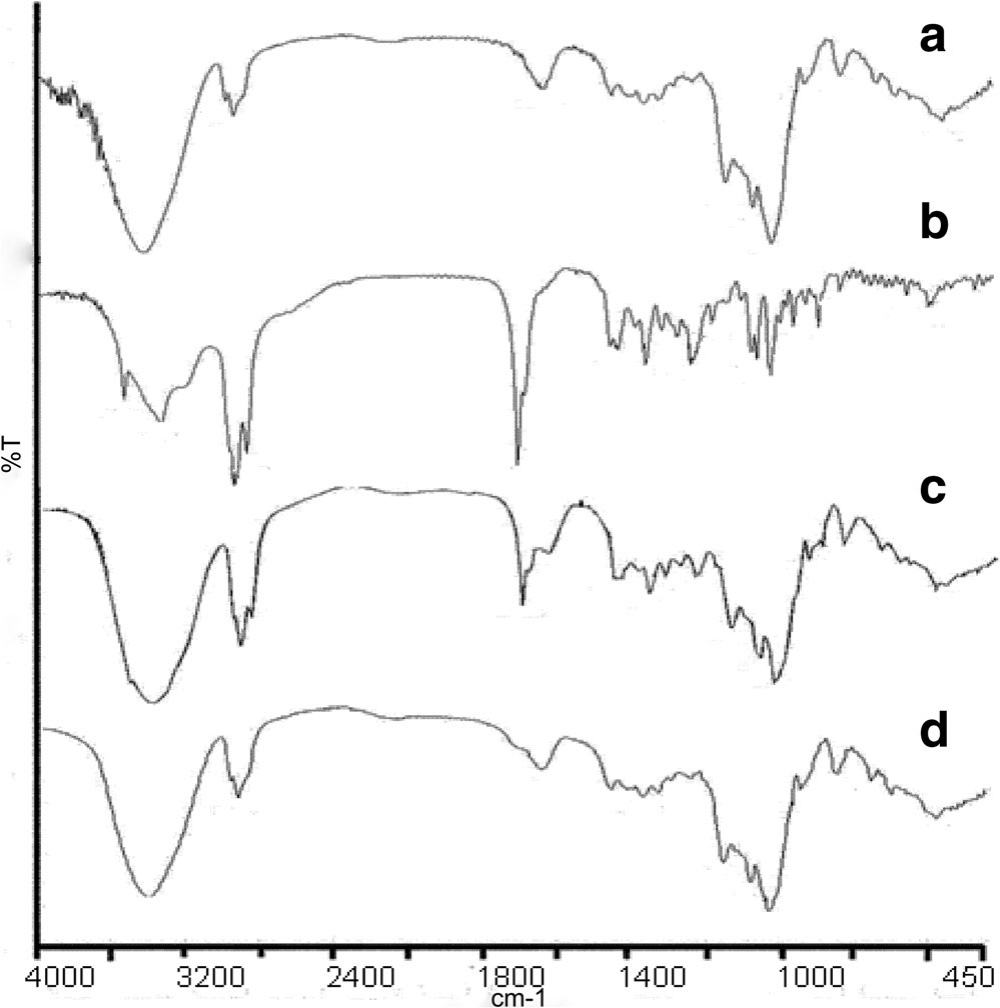
The FTIR spectra of (a) SMEDDS, (b) ZgI, (c) physical mixture, (d) ZgI-SMEDDS

The FTIR spectra of SMEDDS, ZgI, physical mixture, and ZgI-SMEDDS are presented in Fig. 5. As shown in the spectrum of SMEDDS (Fig. 5a), there are absorption peaks at 3421 cm^−1^ (for νO-H stretching vibration), 2932 cm^−1^ (for νC-H stretching vibration), and 1157 cm^−1^ and 1083 cm^−1^ (for νC-H, νC-O stretching vibration). Compared to SMEDDS, ZgI had a group of νC=O, so its spectrum (Fig. 5b) showed a characteristic absorption peak of νC=O at 1715 cm^−1^ (for νC=O stretching vibration). Except the characteristic absorption peak of νC=O, the spectrum of physical mixture (Fig. 5c) was approximately similar to that of SMEDDS. In contrast, the characteristic absorption peak of νC=O has disappeared from the spectrum of the ZgI-SMEDDS (Fig. 5d). Therefore, the characteristic absorption peak of νC=O at 1715 cm^−1^ allowed the distinction between physical mixture and ZgI-SMEDDS. Accordingly, we could conclude that ZgI was dissolved in the SMEDDS and the group of νC=O might be involved in the solution, leading to the absence of νC=O characteristic absorption peak.

#### DSC

**Fig. 6:**
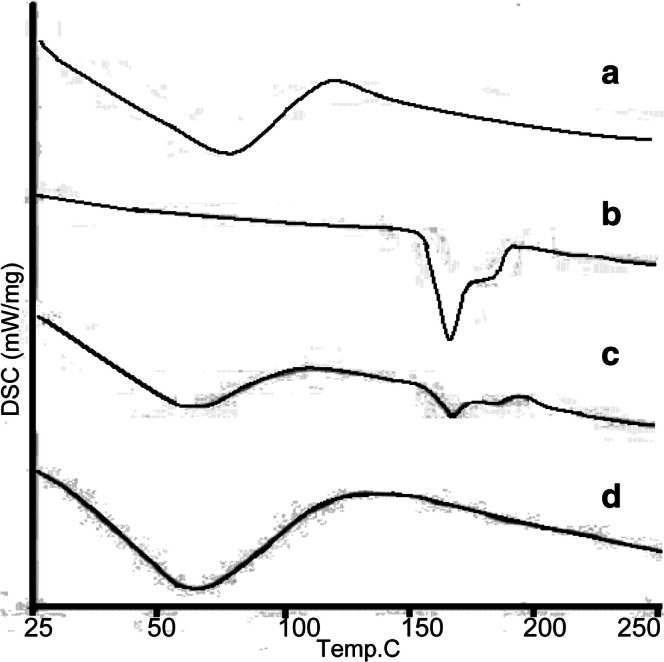
The DSC thermograms of (a) SMEDDS, (b) ZgI, (c) physical mixture, (d) ZgI-SMEDDS

DSC thermograms are shown in Fig. 6. The thermogram of SMEDDS (Fig. 6a) illustrated a very broad endothermic effect, which attains a maximum 86°C, and ZgI is characterized by a sharp melting endotherm at 175°C (Fig. 6b). The DSC thermogram of physical mixture (Fig. 6c) displays two peaks: a broad endotherm at 86°C followed by the endothermal melting peak at 175°C characteristic of ZgI. In the meanwhile, there is a noticeable difference between physical mixture and ZgI-SMEDDS. The characteristic peak of ZgI is absent in the DSC thermogram of inclusion complex (Fig. 6d), which confirms the presence of ZgI in an amorphous form.

#### TEM

**Fig. 7:**
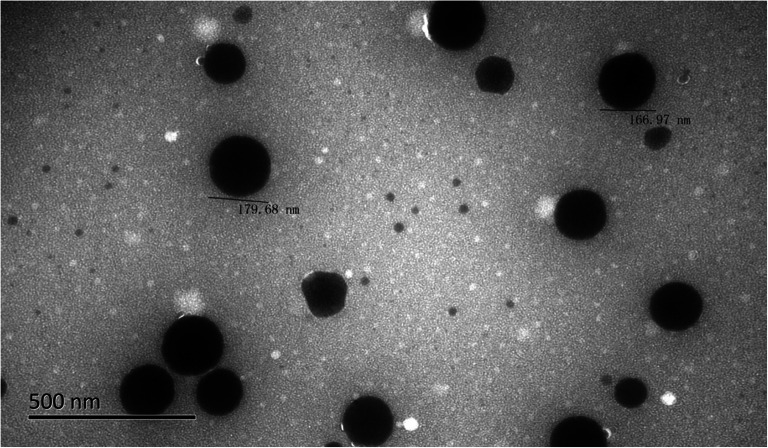
Micromorphology of the ZgI-SMEDDS

ZgI-SMEDDS was appropriately diluted, and it was revealed by transmission electron microscopy that the SMEDDS microemulsion was spherical and uniform in size. The TEM image of ZgI-SMEDDS is shown in the Fig. 7.

### Stability of ZgI-Loaded SMEDDS

The stability data of ZgI-loaded SMEDDS was shown in Table IV. From Table IV, we can conclude that the ZgI-SMEDDS could keep stable at least 3 months without sediment of ZgI in stability chamber. On the two specified storage conditions (25°C/60% RH and 40°C/75% RH), the emulsion was clear liquid all the time, drug content did not decrease, and the particle size did not change.

**Table IV:** Stability Test Result of ZgI-Loaded SMEDDS ($\overline{x}$ ± S, *n* = 3)

Month	Condition	Appearance	Drug content (%)	Particle size (nm)
0	25°C 60% (RH)	Clear liquid	100.02 ± 0.16	206.97 ± 4.35
1		Clear liquid	100.18 ± 0.27	207.11 ± 3.54
2		Clear liquid	100.23 ± 0.19	206.88 ± 4.01
3		Clear liquid	100.08 ± 0.17	208.15 ± 4.75
0	40°C 75% (RH)	Clear liquid	100.19 ± 0.37	207.08 ± 5.61
1		Clear liquid	100.42 ± 0.36	207.22 ± 3.44
2		Clear liquid	100.22 ± 0.30	209.16 ± 3.08
3		Clear liquid	100.11 ± 0.42	206.70 ± 4.82

*RH* relative humidity

### *In Vitro* ZgI Release from SMEDDS

**Fig. 8:**
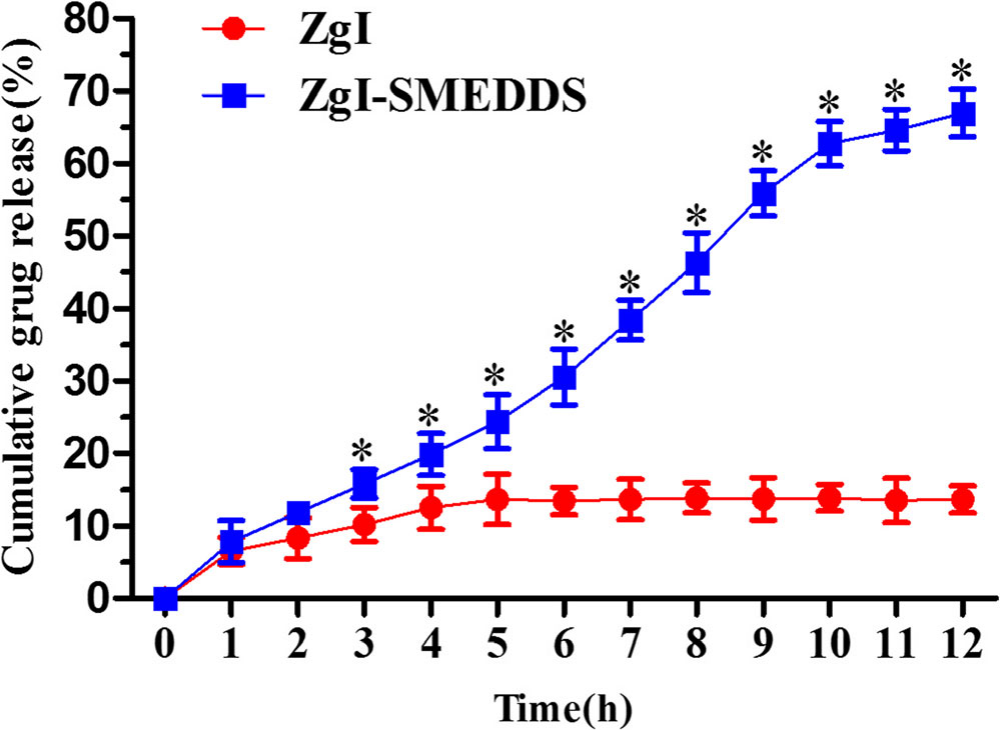
The cumulative drug release of the ZgI-SMEDDS and ZgI. Compared with the ZgI group, *P < 0.05

The *in vitro* cumulative release profile of ZgI from ZgI-SMEDDS is shown in Fig. 8. The *in vitro* cumulative release of ZgI from D-SMEDDS was found to be 78.96 ± 2.83% within 12 h. Compared with the ZgI, ZgI-SMEDDS had a quicker release rate, which showed that the release of ZgI had been greatly improved by the SMEDDS.

### *In Situ* Single Pass Intestinal Perfusion

SD rats were used to study the *in situ* intestinal perfusion of ZgI and ZgI-SMEDDS. The apparent permeability coefficients (*P*_*app*_) of the ZgI and the ZgI-SMEDDS were 1.04 ± 0.02 × 10^−4^ cm/s and 5.71 ± 0.24 × 10^−4^ cm/s, respectively (*P* < 0.05). The drug absorption percentage (*A*%) of the ZgI and the ZgI-SMEDDS were 1.04 ± 0.02 × 10^−4^ cm/s and 5.71 ± 0.24 × 10^−4^ cm/s, respectively (*P* < 0.05). The absorption rate constant (*K*_a_) of the ZgI and the ZgI-SMEDDS were 1.04 ± 0.02 × 10^−4^ cm/s and 5.71 ± 0.24 × 10^−4^ cm/s, respectively (*P* < 0.05). The *P*_app_, *A*%, and *K*_a_ of SMEDDS were significantly increased compared to those of the ZgI.

### *In Vivo* Activity Study

Kunming (KM) mice were used to study the *in vivo* activity of raising white blood cells (WBCs) of ZgI SMEDDS. Mice were injected with cyclophosphamide (200 mg kg^−1^) intraperitoneally to make myelosuppression model. Compared with the normal group, the WBC count in model group was markedly decreased (*P* < 0.05) and the WBC count in ZgI group was markedly decreased (*P* < 0.05) too. Compared with the model group, the WBC count of ZgI-SMEDDS group was significantly increased (*P* < 0.05); compared with ZgI group, the WBC count of ZgI-SMEDDS group was significantly increased (*P* < 0.05). The effect of each test group on the number of peripheral white blood cells in mice is shown in the Table V.

**Table V:** Effect of ZgI-SMEDDS on WBC of Myelosuppression Mice ($\overline{x}$ ± S, *n* = 10)

Groups	Number	Dosage (mg kg^−1^)	WBC (×10^9^)
Normal	10	10	5.31 ± 1.8*^Δ^
Model	10	10	2.23 ± 0.32
ZgI	10	10	2.81 ± 0.15
ZgI-SMEDDS	10	10	4.59 ± 0.27*^Δ^

Compared with the model group, *P < 0.05, **P < 0.01; compared with the ZgI group, ^*Δ*^*P* < 0.05, ^*ΔΔ*^*P* < 0.01. *ZgI z*iyuglycoside I, *ZgI-SMEDDS z*iyuglycoside I–self-microemulsifying drug delivery system, *WBC* white blood cell

### *In Vivo* Pharmacokinetic Study

**Fig. 9:**
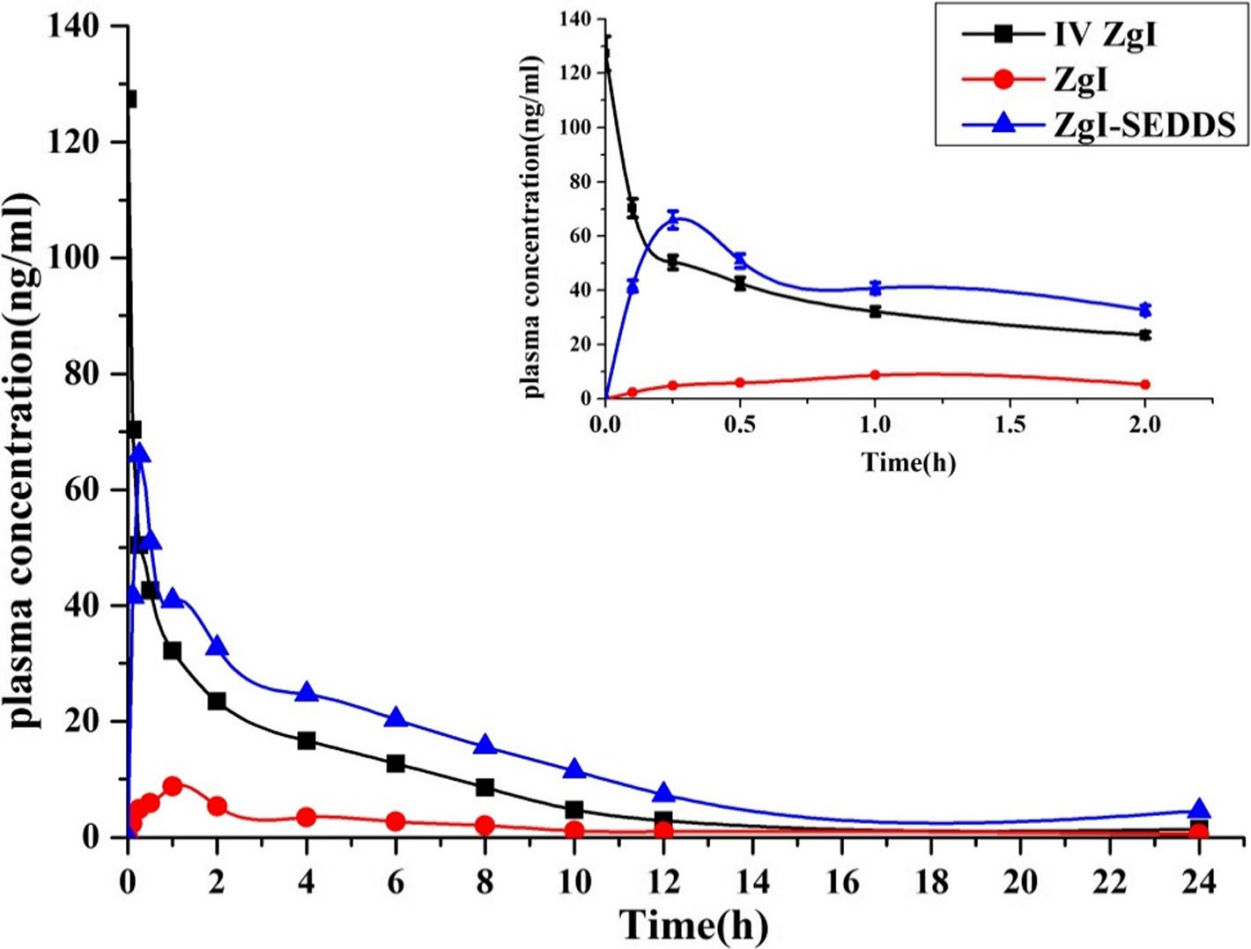
Mean plasma concentration-time curves of ZgI after i.v of a single dose of 2 mg/kg of ZgI Tween-20 solution, oral administration of a single dose of 20 mg/kg of ZgI Tween-20 solution, and equivalent dose of oral ZgI-SMEDDS. Picture on the top right corner was the plasma concentration-time curves for 0-2 h duration (mean ± SEM, *n* = 6/group)

The mean plasma concentration-time curve of ZgI after i.v of a single dose of 2 mg/kg (equivalent to free ZgI) of ZgI Tween-20 solution, oral administration of a single dose of 20 mg/kg (equivalent to free ZgI) of ZgI Tween-20 solution, and equivalent dose of oral ZgI-SMEDDS were shown in Fig. 9. The *C*_max_ of oral ZgI-SMEDDS, oral ZgI, and i.v ZgI were found to be 65.95 ± 8.74, 8.75 ± 1.21, and 127.3 ± 26.87 ng/mL, respectively. The blood concentration of ZgI was dramatically elevated by SMEDDS at 30 min after administration. The pharmacokinetic parameters were calculated using anon-compartment model and were summarized in Table VI. Compared with the ZgI group, the AUC_0-t_ and *C*_max_ of the ZgI-SMEDDS were markedly increased (*P* < 0.05). What’s important is that the oral ZgI-SMEDDS led to a 6.94-fold higher absolute bioavailability (21.94 ± 4.67) % than ZgI (3.16 ± 0.89) %. We also find that the mean resident time (MRT) was increased and clearance (CL) was decreased, which implied that the SMEDDS may prolong the retention time of ZgI *in vivo* and distribute it in different tissues.

**Table VI:** PK Parameters of ZgI (i.v and p.o) and ZgI-SMEDDS (p.o) ($\overline{x}$ ± *S*, *n* = 6)

PK parameters	Units	i.v ZgI	ig (intragastrical administration)	
			ZgI	ZgI-SMEDDS
Dose	mg/kg	2	10	10
*V*_d_	L/kg	189.34 ± 36.47	2983.4 ± 289.27	1214.69 ± 143.57*
CL	L min^−1^ kg^−1^	8.37 ± 1.05	91.51 ± 11.28	25.87 ± 3.56*
AUC_0-t_	ng min mL^−1^	246.55 ± 28.43	78.12 ± 12.46	270.52 ± 37.18*
MRT	h	5.42 ± 0.65	3.26 ± 0.18	6.55 ± 0.26*
*C*_max_	ng mL^−1^	127.3 ± 26.87	8.75 ± 1.21	65.95 ± 8.74
*T*_1/2_	h	12.67 ± 1.33	19.32 ± 4.75	19.2 ± 5.21*
*T*_max_	h	0.01	0.25	1
*F*	%	–	3.16 ± 0.89	21.94 ± 4.67

Compared with the ZgI group, *P < 0.05, **P < 0.01. *i.v* intravenous

## CONCLUSION

We have successfully developed optimal formulations of the ZgI-loaded SEDDS, which can increase the solution rate, solubility, and bioavailability of ZgI. The new emulsion formulations are a promising alternative to oral delivery of water-insoluble drugs like ZgI. Our studies illustrated that SMEDDS containing Obleique CC497, Tween-20, and Transcutol HP with a ratio of 0.25/0.45/0.30 was showed to increase solubility and dissolution rate of ZgI and it may remain stable at least 3 months under limited conditions. The SMEDDS had a faster release rate than plain ZgI. *In vivo* evaluation, we found that SMEDDS showed nearly a sevenfold greater absorption of ZgI compared to the same oral dose of the plain ZgI Tween-20 solution. Finally, the SMEDDS can significantly improve the efficacy of ZgI to increase WBCs. Thus, our studies indicated that the new self-microemulsifying system could be a valuable drug delivery to ZgI with improved oral bioavailability and biological activity.
